# Right pulmonary artery compression following acute type A aortic dissection resulting in acute right ventricular heart failure: A case report

**DOI:** 10.34172/jcvtr.32947

**Published:** 2024-03-13

**Authors:** Hedieh Alimi, Azadeh Izadi Moud, Asal Yadollahi

**Affiliations:** ^1^Vascular and Endovascular Surgery Research Center, Faculty of Medicine, Mashhad University of Medical Sciences, Mashhad, Iran; ^2^Department of Cardiovascular Diseases, Faculty of Medicine, Mashhad University of Medical Sciences, Mashhad, Iran

**Keywords:** Aortic dissection, Pulmonary artery, Right ventricular dysfunction

## Abstract

Right ventricular failure is a mechanism of hemodynamic collapse in acute aortic dissection. Mostly RV failure happens as a result of coronary malperfusion secondary to compression of right coronary artery ostium by the false lumen of type A aortic dissection or the dissection flap involving this coronary artery. Another mechanism is compression of pulmonary artery and an acute rise of pulmonary pressure below the level of obstruction, which is rarely reported. Herein, we presented an 82-year-old man who was admitted with type A aortic dissection in whom echocardiographic examination revealed right pulmonary artery compression resulting in acute right ventricular failure.

## Introduction

 Extrinsic compression of pulmonary arteries is a rare etiology of right ventricular failure.^1^ Anatomical course of the right pulmonary artery after deriving from the main pulmonary artery is posterior to the ascending aorta and its relative position to DAO is anterior and is just anterior to the right main bronchus.^2,3^

 Therefor when aortic dissection occurs at the posterior wall of the ascending aorta, mass effect of the hematoma can compress the right pulmonary artery.^4,3^ The thin-walled RV cannot handle an acute pressure overload which results in rapid RV dilatation and dysfunction.^5^

## Case Presentation

 An 82-year-old man with a history of hypertension, presented to the Emergency Department 8 hours after the sudden onset of a severe chest pain. He had been hospitalized for palpitation and rapid atrial fibrillation ten days earlier and the rhythm had been converted to sinus and he was taking amiodarone, bisoprolol, losartan, atorvastatin, ASA, clopidogrel and nitroglycerin thereafter.

**Figure 1:**
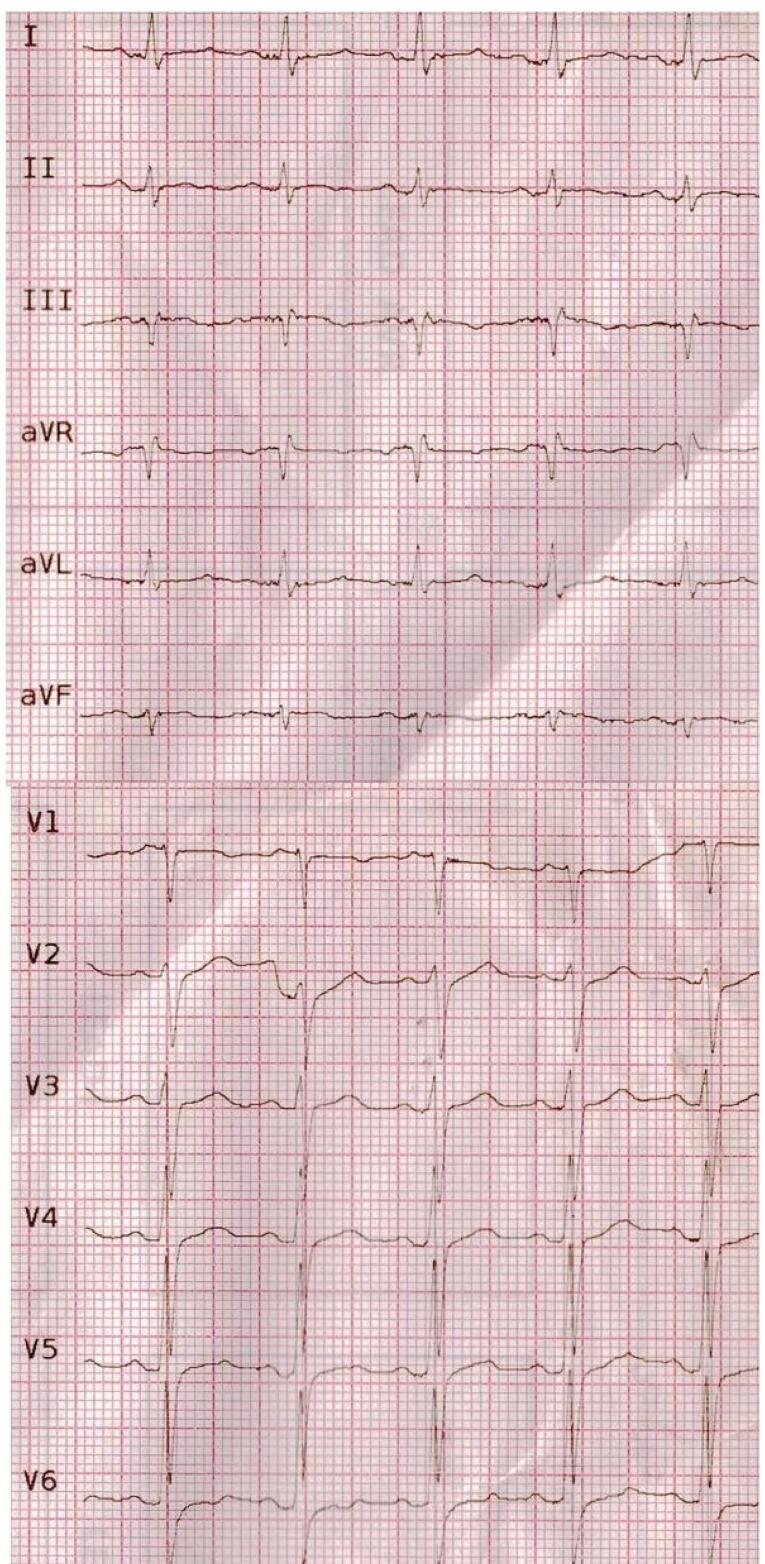
Electrocardiogram showing sinus rhythm without evidence of ST T changes but there is a S1Q3T3 pattern

 On physical examination, his blood pressure was 120/80 mmHg, and heart rate was 80 beats/min. Chest and heart examinations were not remarkable. Peripheral pulses were normal. A 12-lead electrocardiogram showed sinus rhythm with no evidence of significant acute ischemic changes or any evidence of right ventricular hypertrophy but there was evidence of S1Q3T3 (Figure 1). Laboratory data revealed a hemoglobin of 11.1 g/dl, and white cell count of 9,800/L with a normal differential, Creatinine of 1.7 mg/dl, CRP of 139 mg/L, ESR of 84 and negative COVID-19 RT-PCR. Cardiac troponin was negative (TPI < 0.01). The patient was primarily treated with the diagnosis of acute coronary syndrome and heparinization and anti-ischemic therapy had been started.

 As he was admitted in covid-19 era, spiral lung high resolution computed tomography (HRCT) was done which revealed aneurysmal dilatation of the ascending thoracic aorta of 71 mm diameter and focal hyperdense increased aortic wall thickness.

 Transthoracic echocardiography showed normal left ventricle size with mild systolic dysfunction (Ejection fraction: 45%) without left ventricular hypertrophy. D-shaped left ventricle compatible with right ventricular pressure overload was seen. Right ventricle was severely enlarged with systolic dysfunction. There was no evidence of right ventricular hypertrophy which was against chronic processes. Pulmonary valve was normal. There was trace pulmonary insufficiency without valvular stenosis. Right pulmonary artery narrowing was seen due to compression effect of aortic hematoma resulting in systolic turbulency and gradient of 30 mmHg which could be underestimated due to severe right ventricular failure.

**Figure 2:**
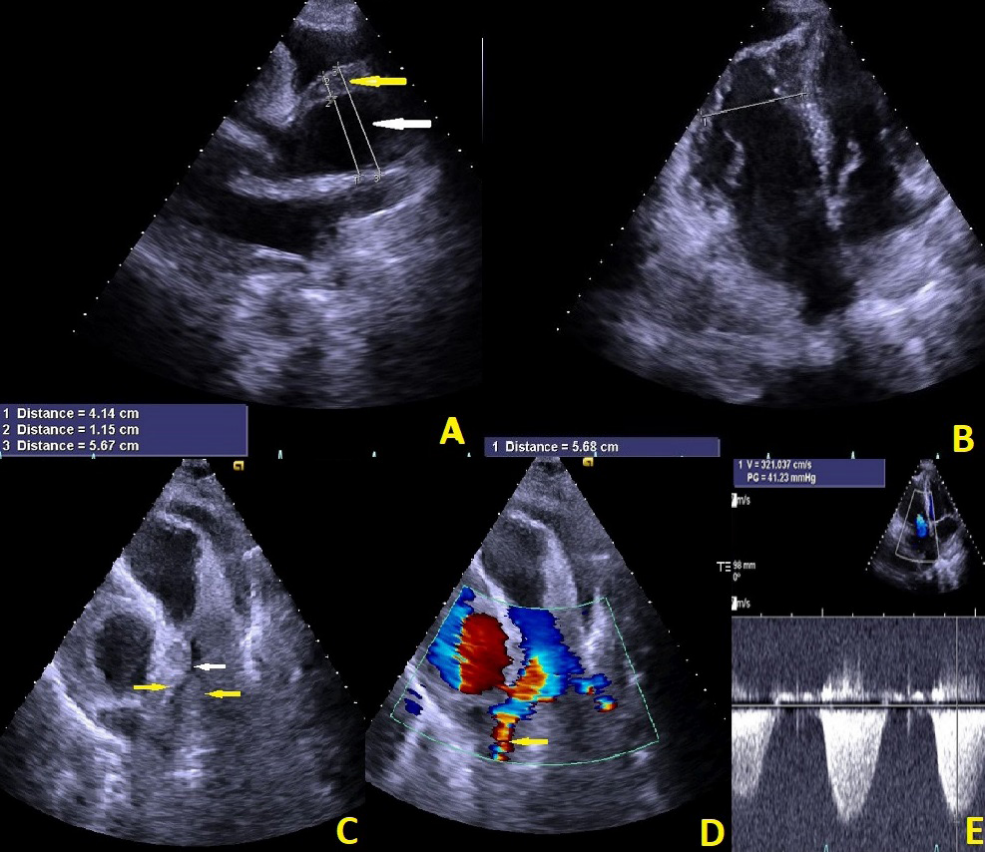
Transthoracic echocardiographic image in parasternal long axis view demonstrates thickened aortic wall from STJ, extended to the distal part of ascending aorta (yellow arrow) indicative of intramural hematoma (A). Markedly dilated right ventricle in four-chamber view (B) Large hematoma around right pulmonary artery (yellow arrows) and right pulmonary artery narrowing (white arrow) (C). Right pulmonary artery narrowing inducing systolic turbulency (yellow arrow) and gradient of 30 mmHg (D and E)

 Tricuspid valve was not stenotic with moderate regurgitation and TRG of 40 mmHg. Pulmonary artery systolic pressure (PASP) based on estimated RA pressure of 15 mmHg was 25 mmHg. Aortic valve was tricuspid with sclerotic changes and mild stenosis and moderate insufficiency. Aortic wall was thickened from STJ, extended to distal part of ascending aorta indicative for intramural hematoma (IMH) (proximal part; (IMH) thickness: 11mm, true lumen: 41mm, total diameter: 56mm) (distal part; false lumen: 19mm, true lumen: 45mm, total diameters: 63mm). No coarctation of aorta was seen. Mild pericardial effusion (8mm) was seen (Figure 2, Movie 1 and Movie 2). After these evaluations aortic dissection was the suspected diagnosis therefore antiplatelet and anticoagulant therapy were discontinued.

**Figure 3:**
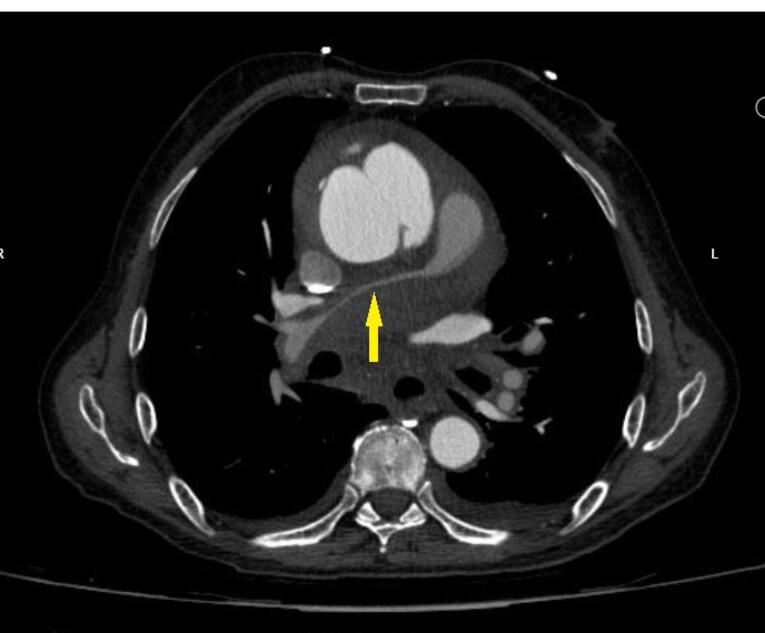
Spiral contrast-enhanced chest CT shows the double lumen aorta with intra-mural contrast, diagnostic for aortic dissection. The main and right pulmonary arteries are compressed by hematoma of type A aortic dissection (yellow arrow)

 For better evaluation of aorta, thoracic and abdominal aortic computed tomography (CT) angiography was done which supported echocardiographic findings. Ascending aorta was dilated with 70mm diameter. The double lumen aorta with intra-mural contrast, confirmed a type A aortic dissection with 14mm thickness of hematoma. Suspicious evidence of contrast extravasation around dissected aorta was seen. Severe compressions of the distal main and right pulmonary artery by hematoma were seen (Figure 3). Emergent cardiac surgery consult was done and the patient underwent surgery in another center. The Bental surgery was done and the diagnosis was confirmed through the surgery. Due to severe right ventricle systolic dysfunction, the patient’s extubation was very difficult. The patient died ten days after the operation due to multiorgan failure.

## Discussion

 Acquired extrinsic pulmonary artery (PA) compression is rarely reported in adults. This condition, while rare, mostly results from mediastinal tumors and non-neoplastic mediastinal pathologies such as infection, inflammatory causes, cyst, aneurysm of thoracic aorta, calcified pericardial ring around PA, and short saphenous venous graft compressing PA. Clinically significant severe PA obstruction is not common in mediastinal tumors because most of them tend to extend laterally. Diagnosis is made through imaging studies which reveal PA compression as displacement of the artery, slit-like lumen and narrowing or cut off of the lumen. Chronic pulmonary flow obstruction can increase right ventricular afterload which secondary results in right ventricular hypertrophy, pulmonary and tricuspid valvular regurgitation and right ventricular failure.^1^

 Few cases have been reported in the literatures about acute PA compression by acute dissecting aorta.

 The same adventitial layer covers pulmonary artery and ascending aorta which limits their mobility against each other. In acute aortic dissection intimal disruption leads to dissection plane in the medial layer of the aortic wall so the dilated false lumen may compress the main and right pulmonary artery. In some cases, adventitial disruption may also occur and blood may encircle the pulmonary artery.^4,3^

 Sudden compression of the pulmonary artery results in an acute rise of pulmonary pressure below the level of obstruction which causes rapid right ventricular dilatation and dysfunction.

 Therefor the clinical manifestations may mimic acute pulmonary thromboembolism and misdiagnosis is possible.^4-6^

 One should consider aortic dissection in the differential diagnosis of patients with chest pain and acute right ventricular failure, especially when their risk factors, symptoms, or findings on examination are compatible with this diagnosis.

 It is very important to make a correct diagnosis because the treatments are different.

 Anticoagulant therapy is essential in acute pulmonary embolism and acute coronary syndrome while it is contraindicated in acute aortic dissection. Electrocardiogram, echocardiography and contrast enhanced chest CT help to confirm the diagnosis.

## Conclusion

 Extrinsic compression of pulmonary vasculature by acute aortic dissection is a rare etiology of acute right ventricular failure.

## Acknowledgments

 We are grateful to Mashhad University of Medical Science for providing general support.

 This research received no specific grant from any funding agency in the public, commercial, or not-for-profit sectors.

## Competing Interests

 The authors declare no conflict of interest.

## Ethical Approval

 This study was approved by Research Ethics Committees of Mashhad University of Medical Sciences (Ethical Code: IR.MUMS.REC.1402.339).

## Funding

 None.

## Supplementary Files

Movie Clip (1): Indicates the Figure number (2). Red arrow shows long axis view and aortic intramural hematoma.

Movie Clip (2): Indicates the Figure number (2). Yellow arrow shows narrowing and white arrow shows systolic turbulent flow of right pulmonary artery because of large hematoma around it.
